# Decaigne on the Effects of Tobacco

**Published:** 1883-02

**Authors:** 


### Decaigne on the Effects of Tobacco.

Most of our readers are familiar with the researches of Decaigne on the effects of tobacco. This gentleman is a member of the Hygienic Society of Paris. Some years ago he published the results of acurate observations on lads and young men, showing that tobacco produced palpitation of the heart, intermittent pulse and anemia, also laziness, stupidity and indisposition to study, besides other evils. Lately the same writer has published the results of an investigation among female smokers, "forty-three cases of which have come under his observation since 1865, when he commenced this special series of studies. Besides disturbance of the digestive function which was common to them all, eight presented a marked intermittency of the pulse without organic disease of the heart. No medical treatment proved of the least avail to correct the distempered function, tonics and sedatives being equally powerless. At length he was compelled to insist on his patients discontinuing the use of tobacco, and in each case where smoking was actually given up—the cautious writer says actually because he found women are more inclined to deceive than men in this regard—the trouble was immediately relieved and ameliorated; and when the suppression of the habit was persevered in for a few weeks with steady purpose, the alarming symptom disappeared altogether. M. Decaigne offered no rationale of the action of the narcotic, and enters into no analysis of the disease now familiar to popular parlance as smoker's heart; but here his observations are supplemented by those of a careful microscopic observer, who has discovered that all narcotics—opium and its preparations, hasheesh, etc., as well as tobacco—act in a peculiar manner upon the colored corpuscles of the blood, producing the phenomenon styled crenation. A few such crenated corpuscles, in the proportion of one to three hundred and fifty, occur in the circulation of persons in normal health, not addicted to narcotics; but in the opium and tobacco habits, when of long standing, the ratio is sometimes as high as one degenerated corpuscle to ten healthy ones, and often attains the figure of one to twenty-five or thirty. In such cases the countenance is pale and almost cyanotic; dark circles appear beneath the eyes, which lack lustre and are deeply sunken, and the respiration is weak and easily disturbed; while the heart palpitates violently upon very slight muscular exertion."

